# Energy-Degenerate Photon-Pair Generation from Individual CsPbBr_3_ Quantum Dots

**DOI:** 10.1021/acs.nanolett.5c02608

**Published:** 2025-09-01

**Authors:** Chenglian Zhu, Leon G. Feld, Simon C. Boehme, Ihor Cherniukh, Maryna I. Bodnarchuk, Maksym V. Kovalenko, Gabriele Rainò

**Affiliations:** † Institute of Inorganic Chemistry, Department of Chemistry and Applied Biosciences, ETH Zürich, CH-8093 Zürich, Switzerland; ‡ Laboratory for Thin Films and Photovoltaics, Empa − Swiss Federal Laboratories for Materials Science and Technology, CH-8600 Dübendorf, Switzerland

**Keywords:** photon-pair generation, single-particle spectroscopy, biexciton cascade, CsPbBr_3_ quantum dots

## Abstract

Beyond single-photon emission, generating correlated *N*-photon bundles, e.g., a photon pair, is essential for various quantum technologies including quantum teleportation and metrology. A widely explored approach exploits the radiative biexciton cascade in individual (mainly epitaxially grown) quantum dots (QDs). Here, we investigate such a cascade in colloidal CsPbBr_3_ QDs, a scalable and solution-processable quantum-light emitter. By matching their size-dependent biexciton binding energies to their size-independent phonon energies, we demonstrate the generation of time-correlated and energy-degenerate photon pairs in large (>15 nm) QDs. Under pulsed excitation at 4 K, we observe pronounced photon bunching, with a g^(2)^(0) of up to 7 in Hanbury Brown and Twiss measurements. The excitation-density-dependent bunching is quantitatively reproduced by multicolor numerical calculations, suggesting the cascade involving biexciton and phonon-mediated exciton decay as origin of the photon pair. Our findings provide new insights into energy-degenerate photon-pair generation in these highly engineerable quantum-light emitters, marking important steps toward their application in quantum-information technologies.

## Graphical abstract

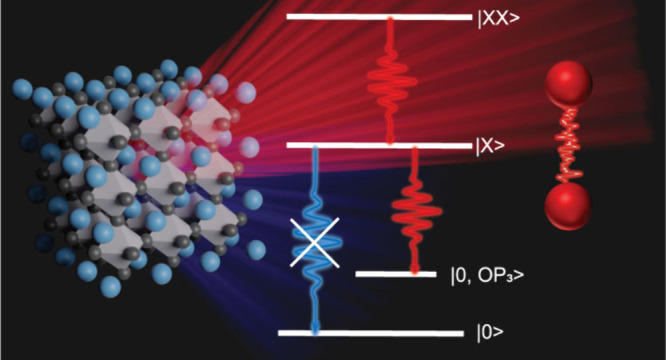

Quantum-light sources producing nonclassical states of light are important building blocks for quantum-information technologies like quantum computing and quantum communication.^1–4^ The underlying processes, including generation and control of on-demand single photons or correlated *N*-photon bundles, are the subject of intense research in quantum-related materials science and device engineering. For instance, epitaxially-grown^5–8^ and colloidal^9–14^ semiconductor quantum dots (QDs), both effective two-level systems, are able to emit a stream of indistinguishable single photons from the radiative recombination of the exciton state (|*X*⟩) to the ground state (|0⟩), as sketched in Figure 1a.

**Figure 1:**
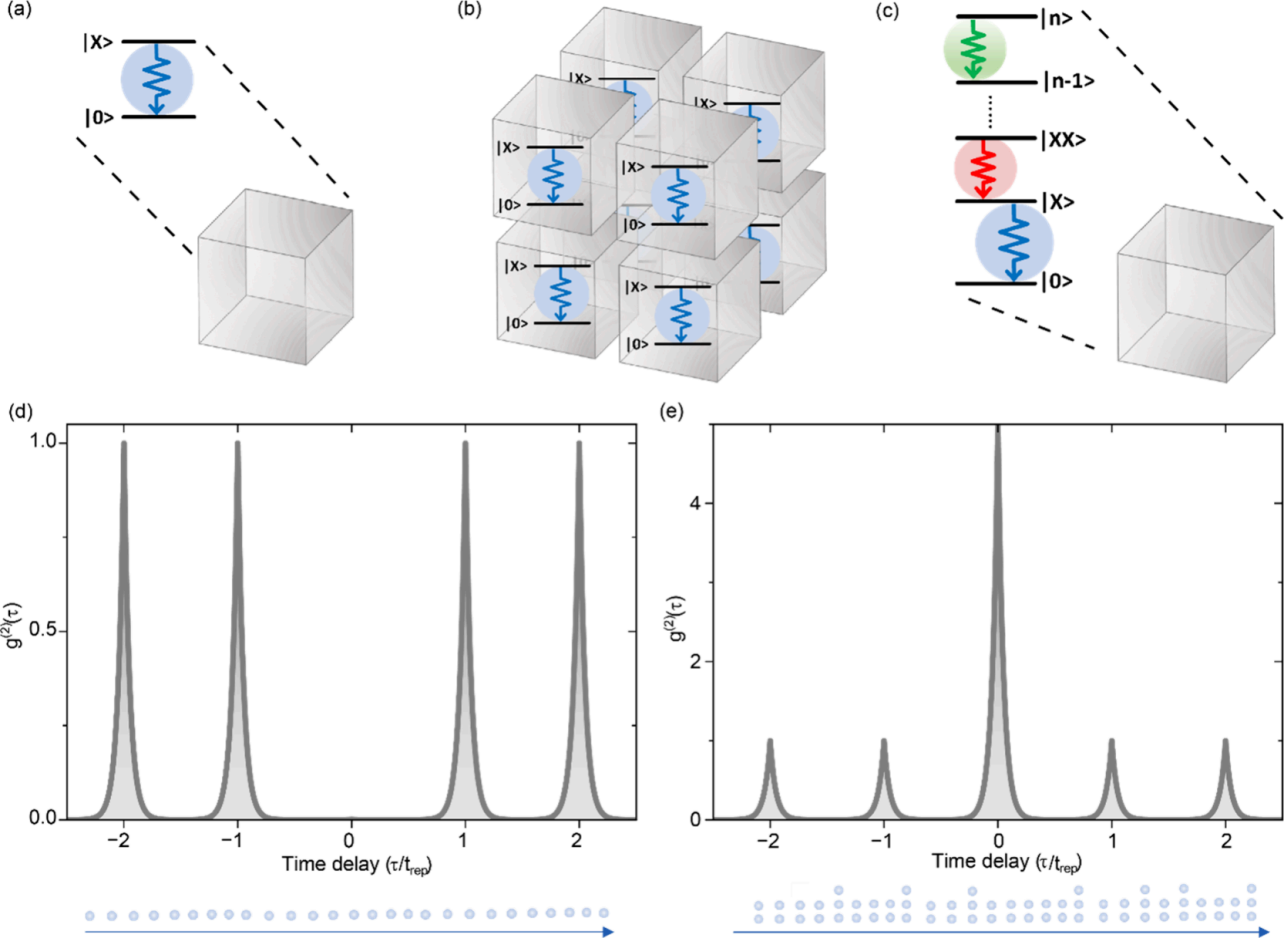
Generation and characterization of single photons and *N*-photon bundles. (a) Single-photon generation in an individual quantum emitter, e.g., a semiconductor QD, representing a two-level system with radiative decay (blue downward arrow) from a singly excited state (|*X*⟩) to the ground state (|0⟩). (b, c) *N*-photon bundles can be generated by coupling multiple QDs through, e.g., a superfluorescence process (b) or in individual QDs through a cascade emission process (c) involving higher-order excited states |*n*⟩, such as the biexciton state |*XX*⟩ ≡ |*n* = 2⟩. (d, e) Exemplary second-order correlation function *g* ^(2)^(τ) of a single-photon (d) and *N*-photon source (e) upon pulsed excitation with a laser repetition rate 1/*t*_*rep*_, showing antibunched and bunched photon statistics, respectively.

Additionally, QDs can serve as an excellent platform for generating *N*-photon bundles (*N* ≥ 2). One explored approach involves emission from several coupled QDs, as sketched in Figure 1b. This method has been demonstrated for a few coupled cavity-embedded QDs^15^ and for QD superlattices displaying superfluorescence.^16–18^ Alternatively, *N*-photon bundles can also be produced from a multiply excited QD if the decay from its n-exciton cascade is (at least partially) emissive,^19–26^ as depicted in Figure 1c. In its simplest form, we look at the biexciton cascade, where |*XX*⟩ represents the biexciton state and |*X*⟩ corresponds to the exciton state, yielding *N* = 2 photons. The perfect correlation in polarization of the photons emitted from the biexciton and exciton state has already enabled the generation of polarization-entangled photon pairs,^22–26^ a vital source for quantum-teleportation technologies.^27^

For both classical and quantum light applications, it is convenient to categorize a light source based on its photon statistics. The latter can be expressed by the second-order correlation function $g^{\left(2\right)}\left(\tau \right)=\frac{\left\langle I\left(t\right)I\left(t+\tau \right)\right\rangle }{\left\langle I\left(t\right)\right\rangle \left\langle I\left(t+\tau \right)\right\rangle }$ , where *I*(*t*) and *I*(*t* + τ) are the detected light intensities at a time *t* and a time τ later, as typically measured in a Hanbury Brown and Twiss (HBT) experiment using a 50/50 beam splitter and two single-photon counting detectors, one in each detection arm. As an important classifying metric, *g* ^(2)^(τ = 0) reflects the likelihood of photon-detection events occurring simultaneously in both detectors. For pulsed excitation, as employed in this work, *g* ^(2)^(0) is determined as the intensity ratio of the zero-delay peak to subsequent peaks. A coherent classical light source like a laser featuring Poissonian photon statistics exhibits the same *g* ^(2)^ peak intensity at all delay times, i.e., *g* ^(2)^(0) = 1. In contrast, quantum-light sources display distinctly different photon statistics: as sketched in Figure 1d and e, a single-photon source exhibits a pronounced dip (antibunching; *g* ^(2)^(0) ∼ 0) at zero-time delay and an *N*-photon source shows a pronounced peak (bunching; *g* ^(2)^(0) ≫ 1).

Among different quantum-light emitters, colloidal CsPbBr_3_ perovskite QDs are emerging as a promising material platform^28–32^ due to their facile synthesis and excellent optoelectronic properties. The well-established and scalable synthesis can produce solution-processable QDs with fine control over their sizes across a wide range from 3 nm^30^ to 30 nm^33^ in a cost-effective way, further allowing the systematic engineering of the desired optical properties. Moreover, the combination of near-unity photoluminescence (PL) quantum yield (QY) and high oscillator strength renders them one of the brightest cavity-free emitters known to date, from room to cryogenic temperature. Additionally, the sub-100 ps radiative decay at 4 K allows for coherent single-photon emission^34–37^ and good indistinguishability^38^ even in the absence of an optical resonator.

Despite the extensive discussion and exploration of single-photon emission in perovskite QDs, the photophysics of photon-pair generation through the biexciton cascade remains poorly explored in this material system. An inherent challenge in this regard is the typically nondegenerate energies of photons from the biexciton cascade, arising from significant Coulomb interaction among charge carriers in these QDs. In epitaxial QDs, one explored solution toward producing energy-degenerate photon pairs has relied on tuning the exciton fine structures on resonance with the biexciton binding energies,^22^ although the tight resonance condition is typically fulfilled only by a small (<1%) and randomly occurring fraction of QDs in the ensemble.^22^ Unfortunately, such a concept is not applicable for perovskite QDs due to their sub-meV fine-structure splitting^39–41^ and typically >15 meV biexciton binding energies.^41–45^

In this work, we propose an alternative route, applicable to perovskite QDs: we exploit the vast synthetic engineerability of CsPbBr_3_ QDs and report the generation of energy-degenerate photon pairs in individual >15 nm CsPbBr_3_ QDs at 4 K by involving one optical phonon (OP_3_) at 19.0 meV (arising from the octahedral stretching)^41,46,47^ in the ground state. The temporal correlation of the photon-pair generated through the cascade process |*XX*⟩ to |*X*⟩ to |0,*OP*_3_⟩ is confirmed by a strong bunching peak in the HBT measurements. Furthermore, the results of the HBT measurements are well corroborated by multicolor numerical calculations, confirming the origin of the photon-pair generation. As a result of the size-dependent Coulomb interaction, fine-tuning of the biexciton binding energies by the QD size realizes the generation of energy-degenerate photon pairs in the newly developed, solution-processable lead halide perovskite QDs.

For the exploration and eventual application of photon pairs generated via the biexciton-cascade process, systems exhibiting a high biexciton QY are essential. In this respect, individual large CsPbBr_3_ QDs (>15 nm, see the TEM images in Figure S1) are a suitable platform for studying photon-pair generation due to their high biexciton QYs.^48–51^ Excitation-density-dependent single-QD PL spectroscopy at 4 K (Figure 2a) identifies the spectral fingerprints of multiexcitons and quantifies their emission efficiencies. With increasing excitation density, spectrally resolved trion (X*) and biexciton (XX) PL emerges to the red side of the exciton band, consistent with previous single-QD spectroscopy studies.^41–44^ With increasing excitation density, the exciton, trion, and biexciton exhibit the expected linear, superlinear, and quadratic dependence, respectively (see inset of Figure 2a), suggesting similarly high QYs for all the emitting species and the absence of excitation-density-dependent activation of nonradiative channels, e.g., the Auger–Meitner recombination. Similar multiexciton QYs are further confirmed via the high g^(2)^(0) values of individual QDs (∼1 in absence of spectral filtering) under low excitation density (with an average number of excitons per excitation pulse of ⟨*n*⟩ ∼ 0.02),^52^ as shown in the upper panel of Figure 2b. The corresponding spectrum is plotted in the top panel of Figure 2c. Given that the temporal separation between subsequent laser excitation pulses (12.5 ns) is much longer than the radiative lifetime of the exciton (∼100 ps^35^), the observed photon statistics (Poissonian) here reflect the combined effects of both the absorption process, which follows Poissonian statistics, and the emission process occurring with similar QY for all the multiexciton species, particularly the exciton and biexciton.^34,35,38,49,52^

**Figure 2:**
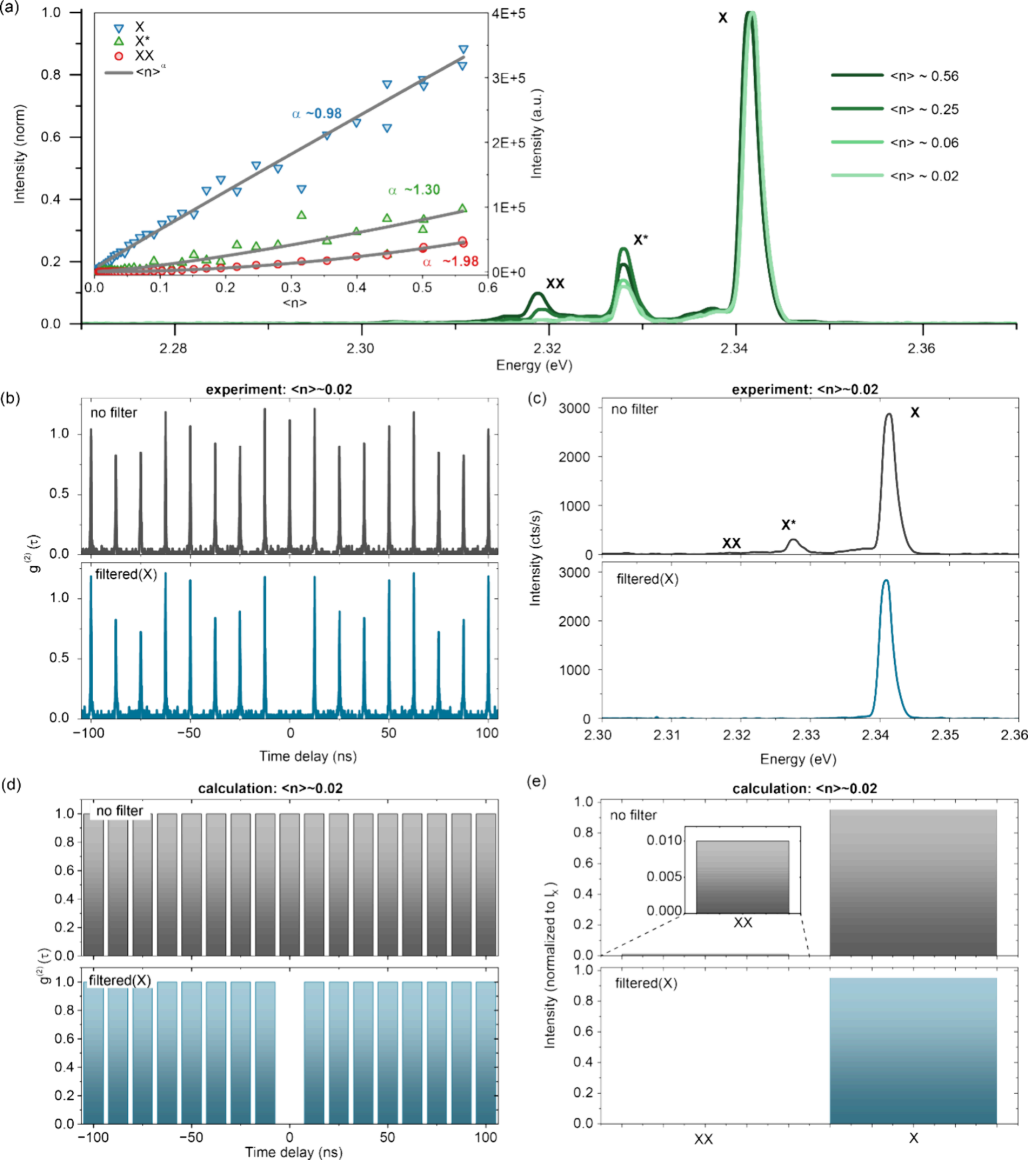
(a) Excitation-density-dependent PL from an individual CsPbBr_3_ QD (16 nm). Trion (X*) and biexciton (XX) emission gradually emerge on the red side of the exciton (X) peak. Inset: Excitation-density-dependent studies of the emission intensity from the exciton (blue downward triangles), trion (green upward triangles), and biexciton state (red circles), respectively, along with power-law fits according to ⟨n⟩^α^, where ⟨n⟩ is the average number of excitons created per excitation pulse. The obtained power-law exponents close to 1, 1.5, and 2 suggest negligible contribution by excitation-density-dependent nonradiative channels such as the Auger–Meitner recombination. (b) Under weak excitation density (⟨n⟩ ∼ 0.02), we observe Poissonian photon statistics for unfiltered emission (upper panel) and single-photon emission from the exciton recombination after spectrally filtering out multiexciton emission (lower panel). The corresponding spectra without and with filters are plotted in (c). (d) Multicolor numerical calculation (adapted from ref 52) of the g^(2)^(0) values without (upper panel) and with (lower panel) spectral filtering. (e) The corresponding calculated intensity of the X and XX emission band.

Earlier studies^34,35,39^ on perovskite QD films suggested near-unity PL QYs at 4 K at the ensemble level. To estimate the PL QY at the single-QD level, we performed excitation-density-dependent studies of the exciton emission peak up to the saturation level, which reliably indicate that most of the studied QDs exhibit a QY exceeding 80%, in agreement with expectations from ensemble measurements (see Section 3.2 in the Supporting Information and Figures S2–S4 for further details). Hence, we can conclude that all the emitting species have very high (near-unity) QYs.

Apart from engineering the photon statistics directly through a suitable design of the intrinsic optical properties of the multiexciton emission, the statistics can also be modified through suitably postselecting emitting photons employing spectral filtering. As shown in the lower panel of Figure 2c, single photons emitted selectively from the exciton-to-ground-state recombination could be obtained with a tunable band-pass filter in the detection path (∼5 meV resolution, for details see Figure S4), hereby efficiently suppressing the detection of any multiexciton photons. As shown in the lower panel of Figure 2b, such emission from the single-exciton band yields a strong antibunching with g^(2)^(0) ∼ 0.02, proving that our QDs remain excellent quantum emitters with discrete energy levels even for large QD sizes significantly exceeding the Bohr diameter.^35^ Similar observations in large CsPbBr_3_ QDs, i.e., g^(2)^(0) ∼ 1 for unfiltered emission and strong antibunching after properly filtering out the multiexciton emission, were also observed in refs 34, 35, and 38 . Conversely, the bunching peak observed in refs 19 and 20 under continuous-wave (CW) excitation reflects the altered photon statistics due to a modified decay rate of the exciton and biexciton with the proposed participation of a dark state. It is worth mentioning that the absence of antibunching cannot be associated with trion emission which is temporally, generally anticorrelated to the exciton emission,^19,40,42,43^ driven by random photocharging events (from the exciton to the trion state) and discharging events (from the trion to the exciton state). In fact, single-photon emission is also observed from these charged species, as attested by a clear antibunching behavior at the trion emission band, as shown in Figure S7.

To further corroborate and rationalize our experimental observations, we performed numerical simulations with a methodology adapted from ref. 52 and considering the detection efficiency with various losses from the source to the detector (see Section 4 in the Supporting Information for more details). As discussed above, we consider the statistics of absorption and emission processes independently under pulsed excitation. In the calculations, the average number of excitons generated per excitation pulse (⟨*n*⟩) is calculated based on the excitation density at the focal plane, the intrinsic absorption cross section^53^ of CsPbBr_3_, and the mean size of the QD sample (derived from the TEM images in Figure S1). Additionally, a factor η_*n*_ is introduced for each step of the emission cascade, representing the probability of emitting one photon during the transition from |*n*⟩ to |*n* – 1⟩. While η_*n*_ may be inherently lower than 100% in case of a nonunity PLQY per transition, η_*n*_ can intentionally be further reduced via postselection approaches such as spectral or polarization-dependent filtering.

In our simulation, the experimentally observed Poissonian statistics of unfiltered light (see the upper panel of Figure 2b) can be reproduced by assuming η_*n*>0_ = 1 (see the upper panel of Figure 2d), consistent with a high intrinsic biexciton PLQY in such large QDs. Likewise, the observed strong antibunching upon filtering (see the lower panel of Figure 2b) can be reproduced by η_*n*=1_ = 1 and η_*n*>1_ = 0 (see the lower panel of Figure 2d), with η_*n*>1_ = 0 mimicking the effect of the applied spectral filter suppressing any multiexciton emission. Moreover, for weak excitation density (⟨*n*⟩ ∼ 0.02) and without spectrally filtered detection, the calculated intensity from the XX is, as expected, rather low compared to that from the X, see the upper panel of Figure 2e. The intensity ratio of the XX to X band is roughly ∼1% in this case, aligning well with the experimental spectra which lacks any clear signature of XX-derived emission under such excitation and detection condition; see the upper panel of Figure 2c.

Most recent studies on generating a stream of temporally correlated photon pairs have focused on epitaxial QDs and utilized a resonant two-photon absorption technique.^22–26^ By altering the absorption statistics, this technique boosts photon emission from biexcitons rather than from excitons. In this work, we employ an alternative method to alter the photon statistics by utilizing only the phonon replica of the exciton as the second photon in the cascade. CsPbBr_3_ QDs possess several optical-phonon modes coupling to the electronic transition arising from octahedral tilting and stretching. Experimentally, sharp and well-defined PL bands from optical-phonon replicas have been reported,^41,46,47^ predominantly involving modes with size-independent energies of about 3.5, 6.3, and 19.0 meV, respectively. We identify these phonon modes as a convenient additional degree of freedom to generate energy-degenerate photon pairs, as illustrated in Figure 3a.

**Figure 3:**
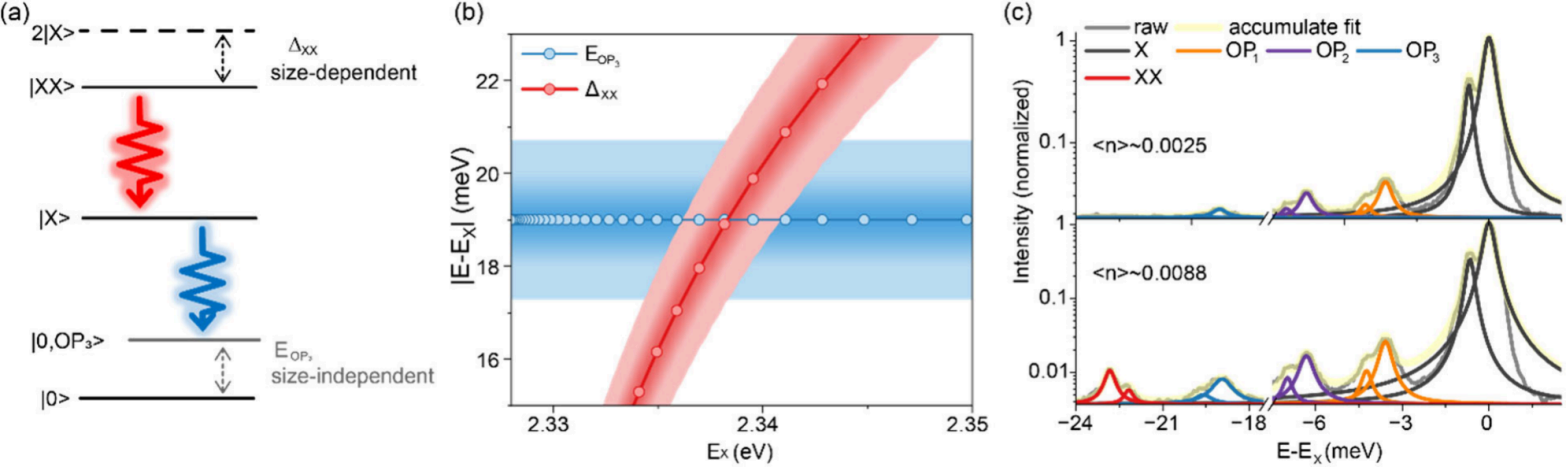
(a) A sketch of the cascade process to generate energy-degenerate photon pairs with one phonon mode coupled to the ground state of the cascade. (b) The binding energies of XX (theoretical curve adapted from ref 42) and energy of the OP_3_ phonon mode (mean values from ref 46) as a function of the exciton energy. In large (>15 nm) CsPbBr_3_ QDs, which emit at around 2.335–2.341 eV, the PL from the |*XX*⟩ to |*X*⟩ transition (red trace) and from the |*X*⟩ to |0, OP_3_⟩ transition (blue trace) are quasi-resonant. (c) PL spectra of a single CsPbBr_3_ QD under low (top panel) and higher (bottom panel) excitation densities, exhibiting spectral fingerprints of the X, three OP modes, and XX, all with doublet fine-structures.

Figure 3b shows the size-dependent biexciton binding energies (red area), about 16–23 meV^41–44^ for the large (>15 nm) CsPbBr_3_ QDs employed here. For suitably sized QDs, the biexciton energy is resonant with the phonon mode (blue area) at 19 meV.^41,46,47^ In this plot, the phonon energy is from the experimental observations reported in ref 46, and the size-dependent biexciton binding energies are from the calculation reported in ref 42. Spectral fingerprints of the biexciton and the phonon replica are plotted in Figure 3c, where we specifically choose an off-resonant case to show the resolved fine structures of the exciton, phonon replicas of the exciton, and biexciton. The doublet exciton (X, black trace) and its corresponding phonon replicas at 3.5 meV (OP_1_, orange trace), 6.3 meV (OP_2_, purple trace) and 19.0 meV (OP_3_, blue trace) are well resolved at ⟨*n*⟩ ∼ 0.0025, where the phonon replicas exhibit the same fine structures as the exciton in terms of relative intensities of the doublet, splitting energies as well as polarization. As the excitation density increases to ⟨*n*⟩ ∼ 0.0088, biexciton PL with a binding energy of 22.8 meV is observed, exhibiting a “mirrored” doublet fine-structure compared to the exciton and its phonon replicas.^19,35,40,42,43^ Examples of the resonant cases can be found in Figure S5, where one fine structure of the biexciton and the OP_3_ phonon, having the same polarization, is degenerate in energy. Overall, from the QD samples with edge length larger than 15 nm, we have more than 20% of the QDs exhibiting the resonant case (for details, see Section 3.3 in Supporting Information).

Next, we investigate whether there is any temporal correlation between the photons emitted from the |*XX*⟩ to |*X*⟩ to |0, OP_3_⟩ cascade. Similar to the experiments for the exciton emission, we apply a tunable band-pass filter in the detection path for the spectral range of biexciton peak such that we consider only photons from the |*XX*⟩ to |*X*⟩ transition and from the |*X*⟩ to |0, OP_3_⟩ cascade. Practically, HBT experiments were done at an excitation density ⟨*n*⟩ ≥ 0.05, where the red-shifted biexciton emission is resolvable, see Figure 4a. An HBT experiment with proper spectral filtering (red traces in Figure 4a) reveals pronounced bunching with g^(2)^(0) = 7.10 ± 2.54 (see Figure 4b). Details regarding the determination of g^(2)^(0) values can be found in Section 3.1 in the Supporting Information. For this HBT experiment, we measure 208 energy-degenerate photon pairs on the 50/50 beam splitter over a 100 s integration time, see Figure S8. In comparison, a Poissonian source with the same average photon counts would theoretically produce only about 28 photon pairs (for details, see Figure S8).

**Figure 4:**
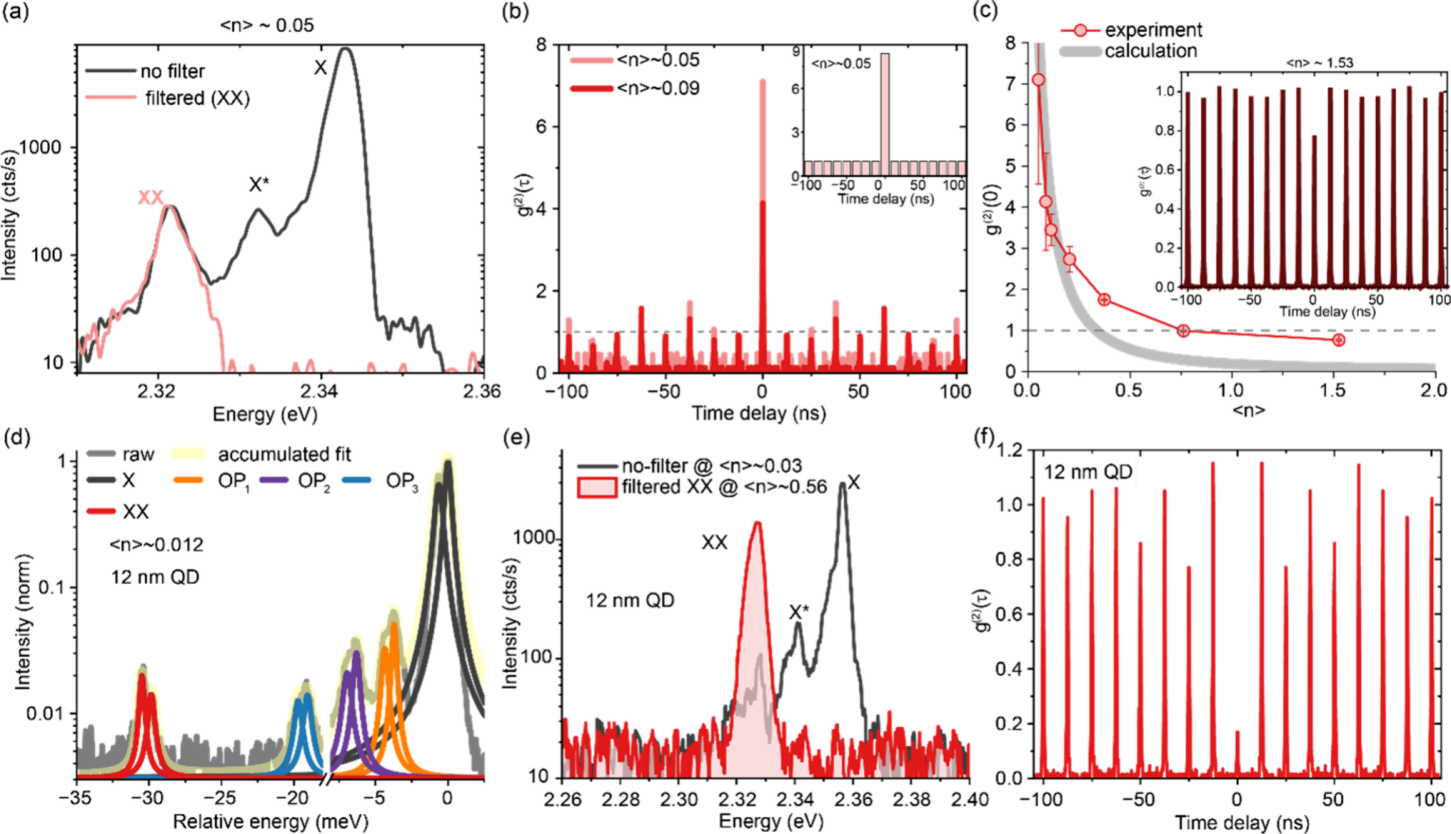
(a) Spectrum of a large (>15 nm) individual CsPbBr_3_ QD at an excitation density of ⟨*n*⟩ ∼ 0.05, where we resolve XX emission. (b) At an excitation density of ⟨*n*⟩ ∼ 0.05, a bunched g^(2)^(τ) is obtained with g^(2)^(0) = 7.10 ± 2.54, attesting the generation of temporally correlated photon pairs. At an excitation density of ⟨*n*⟩ ∼ 0.09, the bunching decreases to 4.13 ± 2.56. Inset: Calculated g^(2)^(0) = 8.39 at an excitation density of ⟨*n*⟩ ∼ 0.05. (c) Excitation-density-dependent g^(2)^(0) values from the experiment (red thin trace with markers) and calculation (gray thick trace). Inset: at an excitation density of ⟨*n*⟩ ∼ 1.53, slight antibunching is observed with g^(2)^(0) = 0.77 ± 0.02. (d) High-resolution spectrum of a 12 nm QD with a doublet fine-structure. (e) Unfiltered spectrum of a single 12 nm QD (dark trace) and filtered XX peak (red trace). (f) The corresponding HBT measurement of the filtered XX peak in (e), which exhibits a clear antibunching g^(2)^(0), attesting pure single-photon emission from the XX to X transition.

In the numerical calculation, we quantify the yield of energy-degenerate photon-pair generation by considering the coupling strength of the relevant optical phonon mode at 19 meV, which we previously reported in ref. 46. In Figure S6, we replot the coupling strength for QDs with an edge length larger than 15 nm as employed in this work. The coupling strength to this 19 meV mode varies from 0.003 to 0.02 across individual QDs, with an average value of around 0.01. We can therefore conclude that roughly 1% of all generated photon pairs from each QD feature energy-degenerate emission by relaxing through the process (|*XX*⟩ → |*X*⟩ → |0, OP_3_⟩) involving such a phonon replica. Calculations employing η_*n*=1_ = 0.01 (the average value of exciton–phonon coupling strength), η_*n*=2_ = 1 (the near-unity biexciton PL QY), and η_*n*>2_ = 0 (zero emission contribution from higher-order multiexcitons) well match the experimental observations: as shown in Figure 4b on the example of low excitation density (⟨*n*⟩ ∼ 0.05), the calculated g^(2)^(0) value (8.39) quantitatively reproduces the measured value (7.10 ± 2.54), within experimental error.

With increasing excitation densities, g^(2)^(0) monotonously decreases, to 4.13 ± 1.18 at ⟨*n*⟩ ∼ 0.09 and to 0.77 ± 0.02 at ⟨*n*⟩ ∼ 1.53, as shown in Figure 4b and c. Further experimental results highlighting the excitation-density dependence are provided in Figure S9. This universal trend of reduced bunching has also been observed in other studies of photon-pair generation,^20,22,54^ due to the probability of individual photon emission (here from |*XX*⟩ to |*X*⟩) surpassing that of the correlated photon pair from the cascade. Consequently, in the high-excitation regime, the photon stream is once again dominated by the single photons emitted from the biexciton recombination, as evidenced by the slightly antibunched g^(2)^(0) values. This experimental trend is also captured by our numerical calculations, shown as the gray trace in Figure 4c. We found excellent quantitative agreement at low excitation densities where the spectra are rather pure and primarily consist of biexciton and OP_3_ emission.

As a final case highlighting the critical role played by the OP_3_ phonon mode in the generation of photon pairs, we conduct experiments on smaller QDs where the energy degeneracy of biexciton binding energy and optical phonon is not fulfilled and where we, hence, do not expect any energy-degenerate photon-pair generation. In QDs with edge length of ca. 12 nm, biexciton emission is red-shifted by more than 10 meV compared to the OP_3_ phonon replica, as plotted in Figure 4d. For such large energetic separations, we can easily isolate the emission of biexciton and the OP_3_ phonon replica by the tunable bandpass filter and then perform HBT measurement selectively using only biexciton emission (red trace in Figure 4e). The corresponding *g* ^(2)^(τ) trace is plotted in Figure 4f, which exhibits a clear antibunching peak for the biexciton emission, attesting pure single-photon emission from the biexciton peak when the OP_3_ phonon is detuned from it. In contrast, for all the large (>15 nm) CsPbBr_3_ QDs employed in the remainder of this work, biexciton and OP_3_ are close enough in energy to pass through the same bandpass filter, producing the observed bunching behavior (see Figure S9).

In conclusion, we report a novel method to generate temporally correlated, energy-degenerate photon pairs in individual large (>15 nm) CsPbBr_3_ QDs at 4 K under pulsed excitation, confirmed via a strong bunching peak with a g^(2)^(0) of up to 7 in HBT measurements. The energy degeneracy is realized via the emission cascade |*XX*⟩ → |*X*⟩ → |0, OP_3_⟩, in which the strongly size-dependent biexciton binding energy can be conveniently matched to the size-independent energy of the optical-phonon mode at 19 meV. With increasing excitation density, the photon statistics gradually transition from bunching to slight antibunching due to the higher probability of generating individual photons rather than correlated photon pairs from the cascade process. Experimental values are rationalized and quantitatively corroborated by multicolor numerical calculations, which account for the absorption and emission statistics independently, confirming the origin of the photon pairs: the phonon-mediated biexciton cascade. Further research may exploit the full potential of this new photon-pair generation scheme, eventually targeting coherent exciton–phonon coupling and photon-pair indistinguishability, thus broadening their potential in quantum-information technologies.

## Supplementary Material



## Data Availability

The data sets generated and/or analyzed during this study are available in the public repository of the ETH Zürich Library (https://doi.org/10.3929/ethz-c-000783044).

## Notes

Synthesis of CsPbBr_3_ QDs; sample preparation for single-QD measurements; details of the experiments; estimation of the PL QY of single QDs; analysis of the high-resolution spectra; spectrally resolved HBT measurements; numerical calculations of the photon statistics (PDF)
