# Supplementary material for: Energy-Degenerate Photon-Pair Generation from Individual CsPbBr3 Quantum Dots
Source: Nano Lett. 2025 Sep 1;25(36):13453–61. doi: 10.1021/acs.nanolett.5c02608 (PMC12426991; doi:10.1021/acs.nanolett.5c02608)
Supplement: Supplementary file 1 [file nl5c02608_si_001.pdf]

Supporting Information

Energy-Degenerate Photon-Pair Generation from Individual $CsPbBr_3$ Quantum Dots

Chenglian Zhu,[1,2] Leon G. Feld,[1,2] Simon C. Boehme,[1,2] Ihor Cherniukh,[1,2] Maryna I. Bodnarchuk,[1,2] Maksym V. Kovalenko[1,2] and Gabriele Rainò[1,2 *]*

[1] Institute of Inorganic Chemistry, Department of Chemistry and Applied Biosciences, ETH Zürich, CH-8093 Zürich, Switzerland

[2] Laboratory for Thin Films and Photovoltaics, Empa – Swiss Federal Laboratories for Materials Science and Technology, CH-8600 Dübendorf, Switzerland

1. Synthesis of $CsPbBr_3$ QDs

Materials

Lead bromide ($PbBr_2$, 99.999%, Sigma Aldrich), cesium carbonate (Cs_2CO_3, 99.9%, Sigma Aldrich), trioctylphosphine oxide (TOPO, min. 90%, Strem Chemicals), oleic acid (OA, 90%, Sigma Aldrich), acetone (≥99.5%, Sigma Aldrich), mesitylene (99%, Thermo Scientific Chemicals), ethyl acetate (99.9%, Sigma-Aldrich), lecithin (>97% from soy, Carl Roth), 1,2-Dioleoyl-sn-glycero-3-phosphoethanolamine (DiOleyl-PEA, >98%, Apollo Scientific).

Synthesis

The $PbBr_2$-TOPO stock solution was prepared by dissolving 367 mg of $PbBr_2$ and 2.15 g of TOPO in 5 ml of mesitylene at 120 °C, followed by cooling down and dilution with 20 ml of mesitylene. The Cs-OA stock solution was prepared by reacting 100 mg of Cs_2CO_3 with 1 ml of OA in 2 ml of mesitylene at 100 °C, followed by cooling down and dilution with 27 ml of mesitylene. The 0.1 M DiOleyl-PEA stock solution was prepared by dissolving 74.4 mg of DiOleyl-PEA in 1 ml of toluene. The 0.13 M lecithin stock solution was prepared by dissolving 50 mg of lecithin in 1 ml of toluene. 15.6 nm and 26.5 nm $CsPbBr_3$ QDs were synthesized by adapting the $PbBr_2$-TOPO approach by Akkerman et al.[1] with a slow injection of Cs-OA and $PbBr_2$-TOPO precursors at higher temperature. For the synthesis of 15.6 nm QDs, 0.85 ml of Cs-OA stock solution and 1.7 ml of $PbBr_2$-TOPO stock solution were slowly simultaneously injected into 1 ml of mesitylene at 60 °C for 8.5 min. Next, 110 µl of the 0.1 M DiOleyl-PEA was added to the crude solution, followed be stirring for 2 min. The QDs were precipitated with 1 ml of acetone, centrifuged for 1 min at 10000 rpm, and redispersed in 1 ml of cyclohexane with 20 µl of 0.01 M DiOleyl-PEA. The QDs were precipitated again with 0.5 ml of acetone, centrifuged for 1 min at 10000 rpm, and redispersed in 1 ml of cyclohexane with 30 µl of 0.01M DiOleyl-PEA. Lastly, the solution was centrifuged for 1 min at 6000 rpm and the supernatant was collected. For the synthesis of 26.5 nm QDs, 2 ml of the Cs-OA stock solution and 4 ml of the $PbBr_2$-TOPO stock solution were simultaneously injected into 0.5 ml of mesitylene at 60 °C for 40 min. Next, 0.5 ml of 0.13 M lecithin was added to 3 ml of the crude solution, followed by stirring for 2 min. The QDs were precipitated with 3 ml of ethyl acetate:acetone mixture (2:1, v/v), centrifuged for 1 min at 12100 rpm, and then redispersed in 1.5 ml of toluene. The solution was centrifuged for 1 min at 4300 rpm and the supernatant was collected.

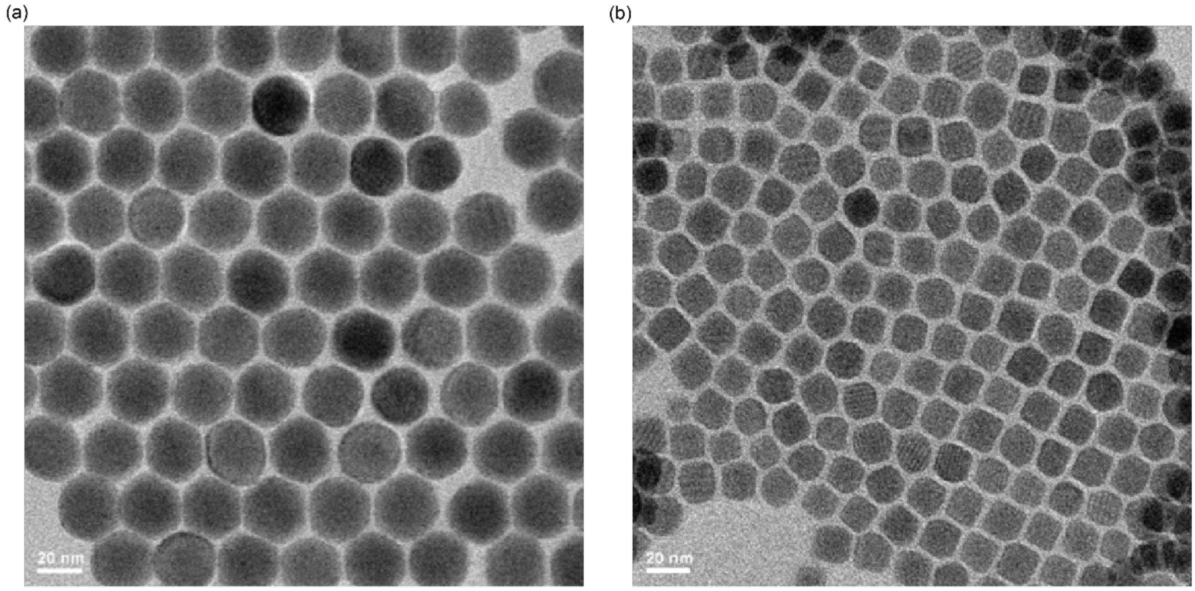

Figure S1. TEM images for the $CsPbBr_3$ QDs with a mean size of 26.5 nm (a) and 15.6 nm (b).

2. Sample preparation for single-QD measurements

The following steps were performed in a glovebox that is kept under nitrogen atmosphere and employing dry and filtered toluene (Acros Organics, 99.85% extra dry over molecular sieves). Original QD solutions (1mg/ml concentration) were first diluted by a factor of 80-100, followed by dilution by a factor of 100 in 3 mass-% polystyrene in toluene. Subsequently, 100 μL of this solution were spin-coated onto a Si substrate (coated with 2 μm SiO_2) at 50 rounds per second for 80 s.

3. More details about single-QD measurements

3.1. Details of the experiments

For single-particle spectroscopy, a home-built μ-PL setup is used. The samples were mounted on xyz nano-positioning stages inside an evacuated liquid-helium closed-loop cryostat (MONTANA INSTRUMENTS) and cooled down to a targeted temperature of 4 K. Single QDs were excited by means of a fiber-coupled excitation laser at an energy of 2.585 eV (480 nm) with a repetition rate of 80 MHz (TOPTICA, < 200 fs pulses), which is focused ($1/e^2$ diameter = 2.4 μm) on the sample by a microscope objective (NA = 0.8, 100×). The emitted light is collected by the same objective and passed through a 90:10 beam splitter and a long-pass filter at 500 nm. A monochromator coupled to a back-illuminated CCD (Princeton Instruments) is used for spectra measurements. Spectra are measured with a grating 1800 (or 300) lines/mm, blaze at 500 nm, yielding around 0.25 (or 1) meV spectral resolution.

A Hanbury-Brown and Twiss setup with a 50/50 beam splitter, two APDs and a TCSPC Module (PicoQuant, PicoHarp) is used for second-order correlation ($g^{(2)}(\tau)$) measurements. In our analysis, $g^{(2)}(0)$ values are determined by the peak height ratio ($\frac{A_0}{\sum_{i=-8,\dots,-1,1,\dots,8} A_i/16}$) of the center peak ($A_0$) and the 16 closest side peaks (A_i, $i = -8, -7, \dots, -2, -1, 1, 2, \dots 7, 8$). Errors may come from the variations in the side peak heights, especially under low excitations. By using the standard deviation of A_i and error propagation, we obtained the standard deviation of the corresponding $g^{(2)}(0)$ values, as plotted in Figure 4c. Additionally, for all the HBT measurements, we use 100 s integration time. To have a good spectral filtering on the X and XX, we apply a tunable band-pass filter with 1-2 nm spectral resolution (c.a. 5 meV, see Figure S4a), realized via a combination of a tunable band-pass filter (Figure S2b, Semrock, cut-off wavelength is tunable between 488 nm and 554 nm with a bandwidth of 20 nm) and a tunable short-pass filter (Figure S2c, Semrock, cut-on wavelength is tunable between 496 nm and 565 nm).

3.2. Estimation of PL QY of single QD

To estimate the QY of an exciton in a single QD, we performed excitation-density-dependent studies of the exciton emission peak up to the saturation level. The QY can be calculated as $QY = \frac{X\ intensity\ at\ saturation}{repetition \times \eta_{overall-det}}$, where $X\ intensity\ at\ saturation$ is the photon count rates detected by the APDs at saturation excitation density, $repetition = 80\ MHz$ and $\eta_{overall-det}$ represents the overall detection efficiency of the detector (APD). To quantify the overall detection efficiency of the setup, we simulated the collection efficiency of our objective[2] (Figure S2) and considered the transmission or detection efficiency of all the optical elements, as listed in Table S1. We estimate an overall detection efficiency of $0.0628 \times \eta_{TBP}$, where the transmission efficiency of the tunable bandpass filter (η_{TBP}) may vary from QD to QD and will be quantified for each of the measured QD (Figure S3a).

To illustrate our assessment methodology, Figure S3a-c report a few selected examples of the excitation-density-dependent study, with the displayed count rate referring to detection events at both APDs. The detected average photon count rate at saturation is determined based on the Poissonian statistics for generating one exciton ($n = 1$): $I(x) = I_{sat}(1 - p(0)) = I_{sat}(1 - \exp(-x/B))$, where $p(0)$ is the probability of zero photon absorption events and B corresponds to the saturation excitation fluence (averagely absorbing $N = 1$ photon). Saturated exciton intensity is determined by the nominal overall detection efficiency and the detected

average photon count rate at saturation, the QY can be inferred for individual QDs. The QY statistics across seven individual QDs are plotted in Figure S3d. It is worth noting that this QY estimation method is sensitive to small variations in the experimental conditions. Factors such as fluctuations in excitation density, spectral diffusion, or slight misalignments could change the observed saturation count rate and thereby lead to an uncertain in estimated QY. With this method, the fits yield single-QD PL QYs on the order of unity. We do note that our estimate exceeds 100% PL QY for two out of the seven individual QDs. However, as noted previously, these values are easily understood considering the large uncertainties and fluctuations of this single-QD methodology. Importantly, statistics across the seven individual QDs suggest a PL QY for the X state of close to 100% at the single-QD level, hereby corroborating our earlier coarse estimate. Overall, while this approach is still not capable of determining the QY with very high precision, it indicates that most of the studied QDs exhibit a QY exceeding 80%, in agreement with the expectations from ensemble measurements.

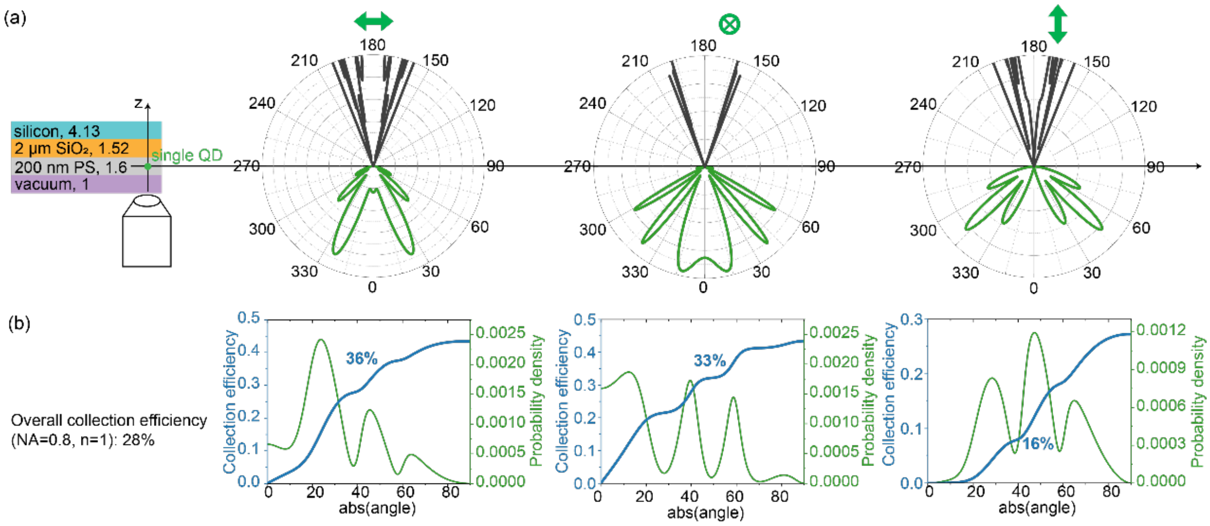

Figure S2. Simulation of the collection efficiency of the objective (NA=0.8), obtained from a near-to-far-field transform model for a light emitter embedded in stratified media and radiating in free-space mode[2]. (a) The stratified media surrounding our single emitter and the far-field angular radiation diagram of the electric dipole along three orthogonal directions. The displayed three dipole orientations account for the crossed transition dipole orientations of the bright triplet exciton, *i.e.*, two in-plane dipoles and one out-of-plane dipole. The emitter radiates both towards the above SiO_2 layer (dark curve) and the bottom vacuum environment (green). (b) Collection efficiency for each transition dipole. The objective collects emission from the lower vacuum side of the stratified structure. On average, we obtained a collection efficiency of 28% for the objective (NA=0.8) used in this work.

Table S1. Determination of the overall detection efficiency.

Efficiency for light at around 530 nm		Nominal values	Reference
Collection efficiency of the objective	$\eta_{coll-obj}$	28%	Simulation in Figure S2
Transmission of the objective	$\eta_{tran-obj}$	95%	Zeiss
Transmission of the tunable band-pass filter	η_{TBP}	50-80%	It varies among QDs, calculated by the exciton intensity before and after applying the filter (see inset of Figure S3a and Figure S4)
Transmission of the 90/10 beam splitter	$\eta_{90/10BS}$	83%	Thorlabs
Transmission of the long-pass filter at 500 nm (#2)	η_{LP}	96%	Thorlabs
Reflection of the mirror (#2)	η_{mirror}	95%	Thorlabs
Transmission of the lens	η_{lens}	90%	Thorlabs
Transmission of the 50/50 beam splitter	$\eta_{50/50BS}$	95%	Thorlabs
Detection efficiency of the APD	η_{APD}	40%	PicoQuant
**Overall detection efficiency**	$\boldsymbol{\eta_{overall-det}}$	$\mathbf{0.0628} \times \boldsymbol{\eta_{TBP}}$	

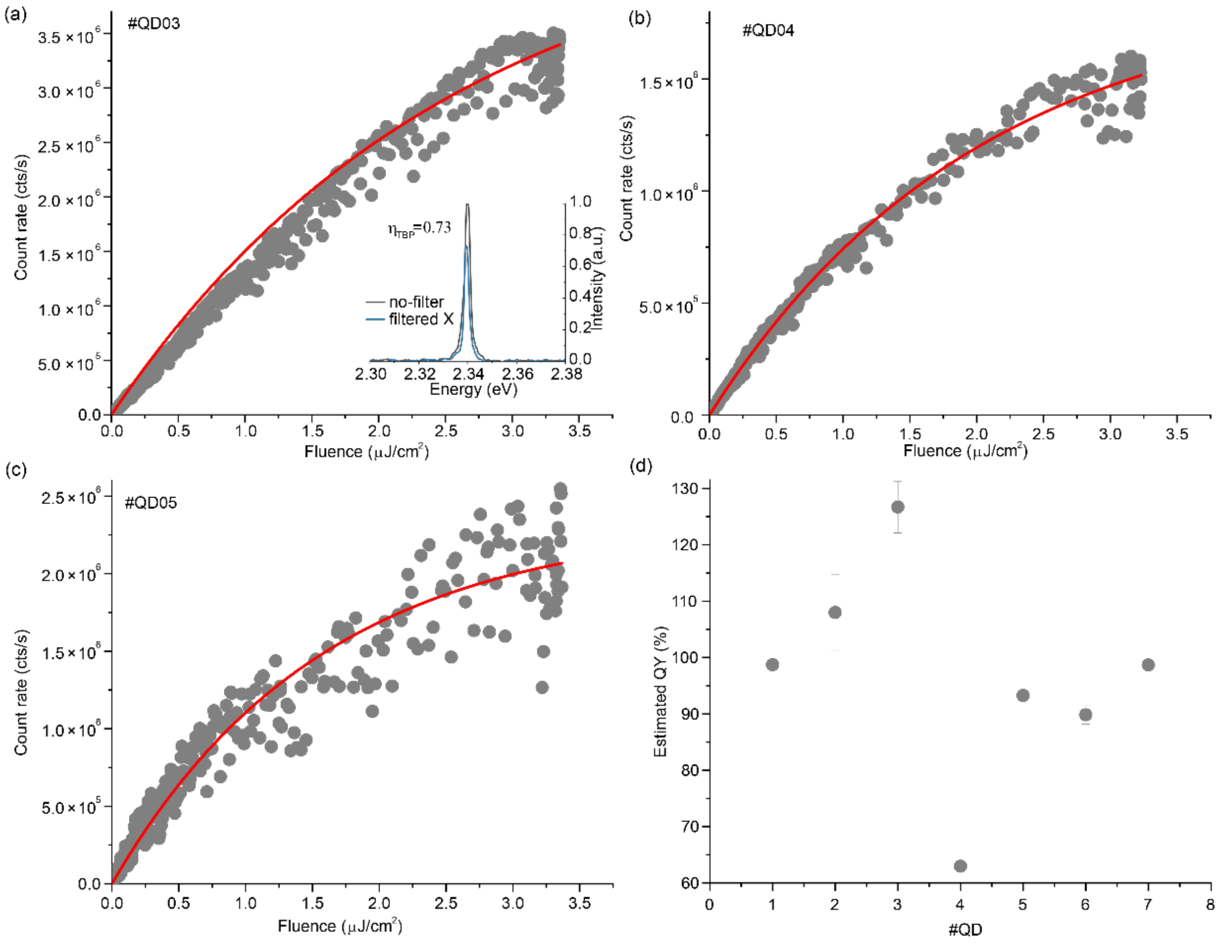

Figure S3. Excitation-density-dependent study on the X emission peak via APDs. (a-c) Three examples of the excitation-density dependent X intensities. (d) Statistics of the estimated QYs.

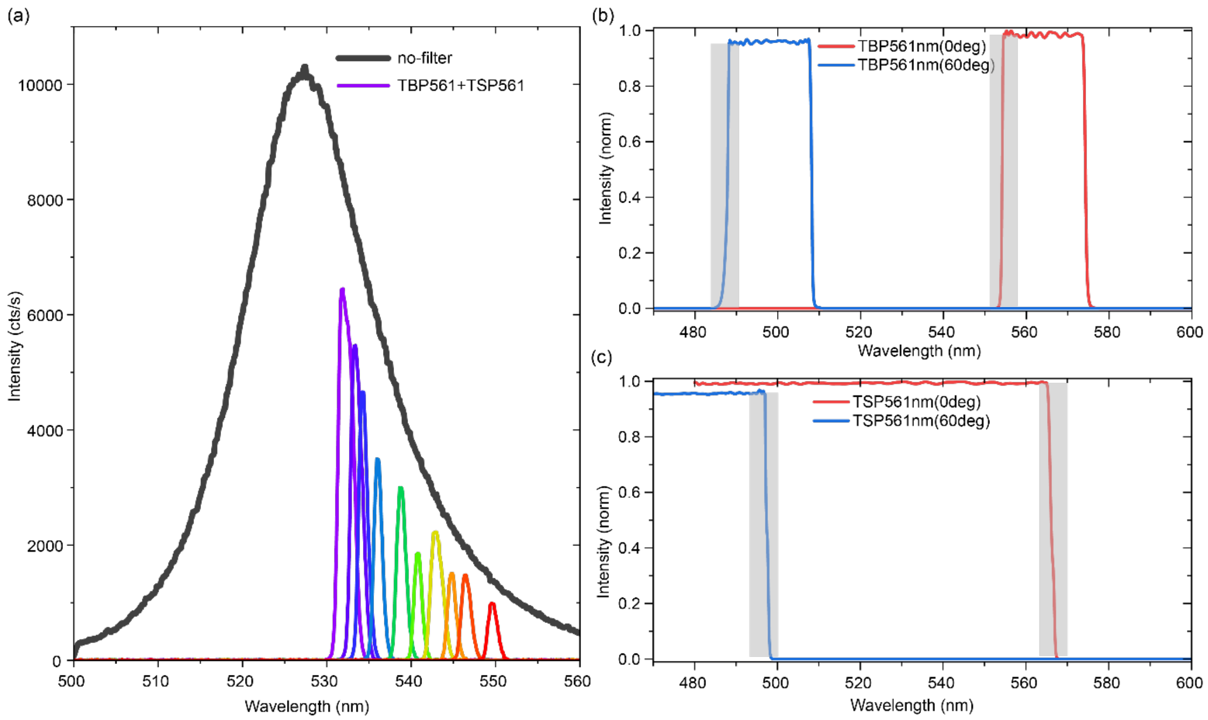

Figure S4. Tunable band-pass filter (~1-2 nm spectral resolution in (a)) realized via the combination of a tunable band-pass filter and tunable short-pass filter, whose angle-dependent spectra are plotted for two exemplary angles (0 and 60 degree) in (b) and (c), respectively.

3.3. Analysis of the high-resolution PL spectra

In perovskite QDs, the bright exciton emission features a triplet arising from the underlying excitonic *fine structure.*[3] In this work, we refer to these three emission peaks as FS_1, FS_2, and FS_3. Since the exciton fine structure is applicable to the emission from the X state, its OP peaks, and the XX state, such labelling applies to the fine structure in all of these emission peaks. To fully resolve the FS peaks of the X band, of the phonon replicas and of the XX band, the entire emission spectrum is fitted with multi-Lorentian functions to extract key features of the various peaks composing the emission spectrum as following:

$$I_{tot}(E) = I_X(E) + \sum_{j=1,2,3} I_{OP_j}(E) + I_{XX}(E) + c \qquad (S1)$$

where $I_X(E)$ is the exciton emission, $I_{OP_j}(E)$ represents different phonon replicas, $I_{XX}(E)$ corresponds to the biexciton emission and c is the background. The exciton term is fitted as follows:

$$I_X(E) = \sum_{i=1,2,3} \frac{2 \times I_{FS_i}}{\pi} \frac{w_{FS_i,X}}{4 \times (E - E_{exc} - \Delta_i)^2 + w_{FS_i,X}^2} \qquad (S2)$$

where $\Delta_i = E_i - E_1$ is the splitting energy between FS peaks. For phonon replicas, we fit as follows:

$$I_{OP_j}(E) = \sum_{i=1,2,3} \frac{2 \times S_j \times I_{FS_i}}{\pi} \frac{w_{FS_i,OP_j}}{4 \times (E - E_{exc} - E_{OP_j} - \Delta_i)^2 + w_{FS_i,OP_j}^2} \qquad (S3)$$

where the S_j is the coupling strength for the phonon mode OP_j. For the biexciton, the fitting follows:

$$I_{XX}(E) = \sum_{i=1,2,3} \frac{2 \times R_{XX} \times I_{FS_i}}{\pi} \frac{w_{FS_i,XX}}{4 \times (E - E_{exc} - \Delta_{XX} + \Delta_i)^2 + w_{FS_i,XX}^2} \qquad (S4)$$

where R_{XX} represents the intensity ratio of biexciton to exciton for the fine-structure state decaying through the same intermediate state. Throught the fittings of different emitting species, the relative intensity distribution and splitting energies among the fine-strucutres are fixed. Examples can be seen in Figure 3c and Figure S5.

In this work, the fraction of QDs yielding the required spectral overlap is a key parameter for quantifying the efficiency of this method in producing "on demand" energy-degenerate photon pairs. To quantify this fraction, we also calculate the energy difference ($\Delta_{pair,FSi}$= $E_{OP_{3,FSi}} - E_{XX_{FSi}}$) between the paired fine-structure states, which go through the same energy sublevels of the exciton for each QD. Since the exciton in perovskite QDs possesses a bright triplet state, we recorded $\Delta_{pair,FSi}$ with the smallest absolute value among the triplet ($\Delta_{pair,absmin}$) for each QD, as plotted in Figure S5c. Error bars represent the spectral resolution accounting for the possible uncertainties during acquisition and fitting. The "energy-degenerate" case is considered when the QDs exhibit $\Delta_{pair,absmin}$ lower than the setup resolution (0.25 meV). Overall, 23% (7 out of 33) of the QDs exhibit a degenerate emission band. However, despite 77% of the QDs do not feature such a perfect spectral overlap, they feature a splitting energy within 3 meV. This implies that all studied QDs (> 15 nm) exhibit a bunching peak when considering the spectral filtering resolution (ca. 5 meV), as experimentally employed for the spectrally resolved correlation measurements shown in Figure S10. In future, the current fraction of QDs satisfying the stringent experimental requirements of energy-degenerate emission bands could be increased by employing QDs from more uniform QD ensembles (*i.e.*, monodisperse in size and shape), thus resulting in reduced spectral variation between the different emitting species.

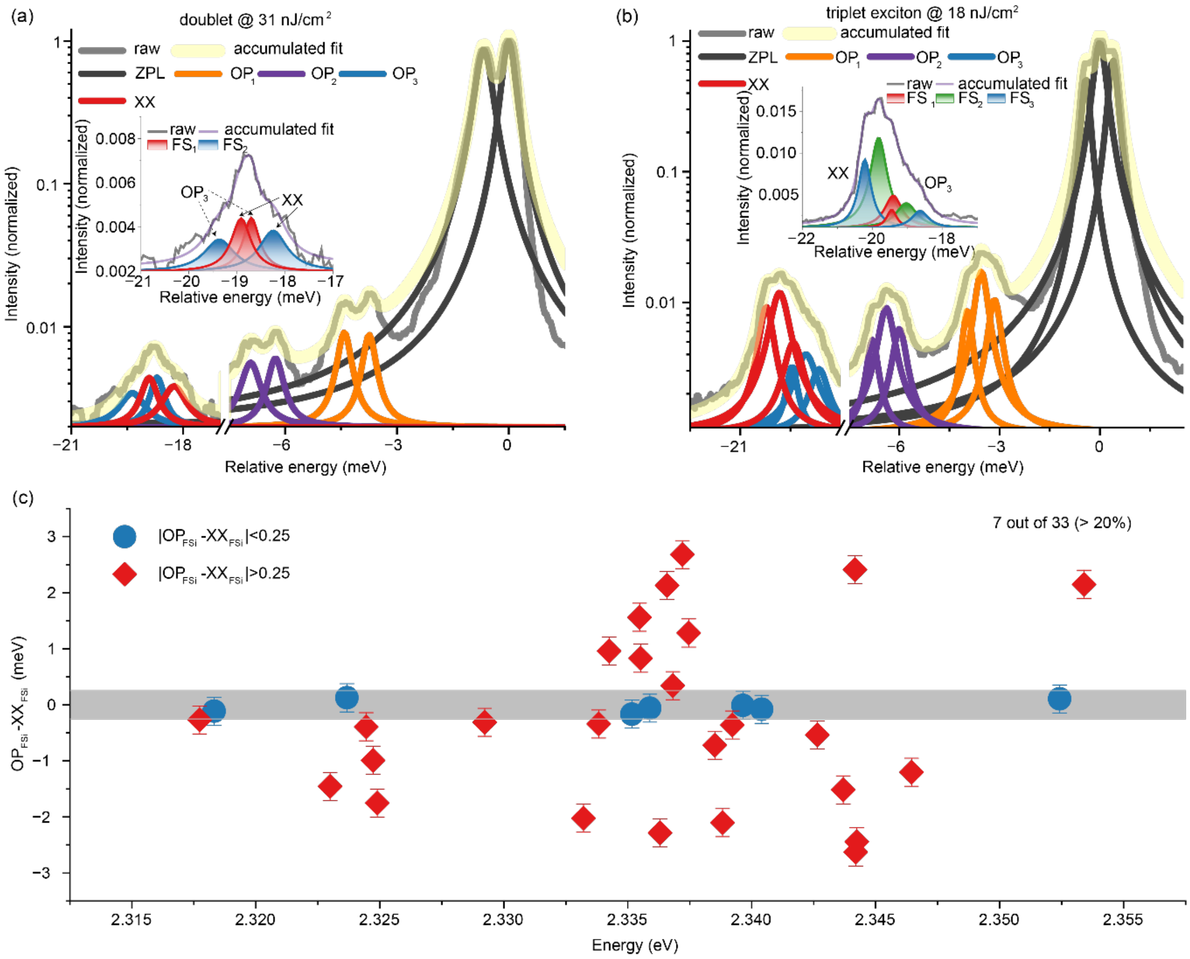

Figure S5. **Energy-degenerate photon pairs.** (a) PL spectrum of a QD with doublet fine-structure (FS), with FS_1 of the doublet biexciton on resonance with the OP_3 phonon. (b) PL spectrum of a QD with a triplet FS, with FS_1 of the triplet biexciton on resonance with the OP_3 phonon. (c) Minimum energy difference between the fine-structure state of the biexciton and the single-exciton OP_3 phonon replica with same polarization. Error bars represent the spectral resolution accounting for the possible uncertainties during acquisition and fitting. For more than 20% of the studied QDs (blue markers), this energy difference is smaller than the spectral resolution of the setup (0.25 meV, grey shaded area) and can, thus, be considered energy-degenerate.

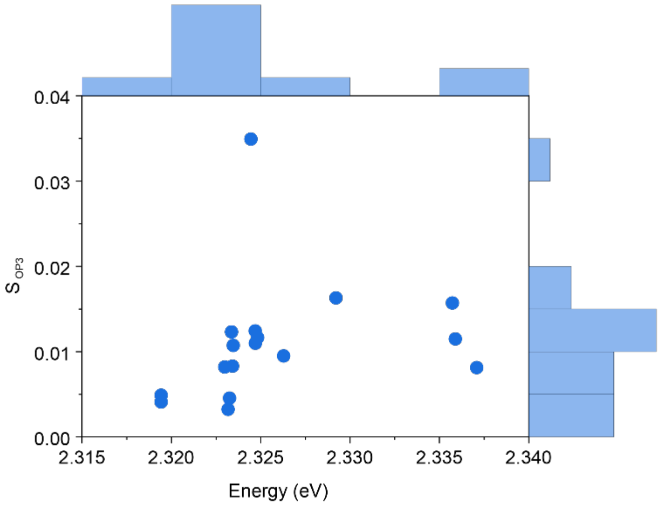

Figure S6. Coupling strength of the phonon replica with phonon energy of 19 meV. On average, the coupling strength (S_{OP3}) of the phonon mode at 19 meV is around 0.01. Values in this plot are taken from ref.[4].

3.4. Spectrally resolved HBT measurements

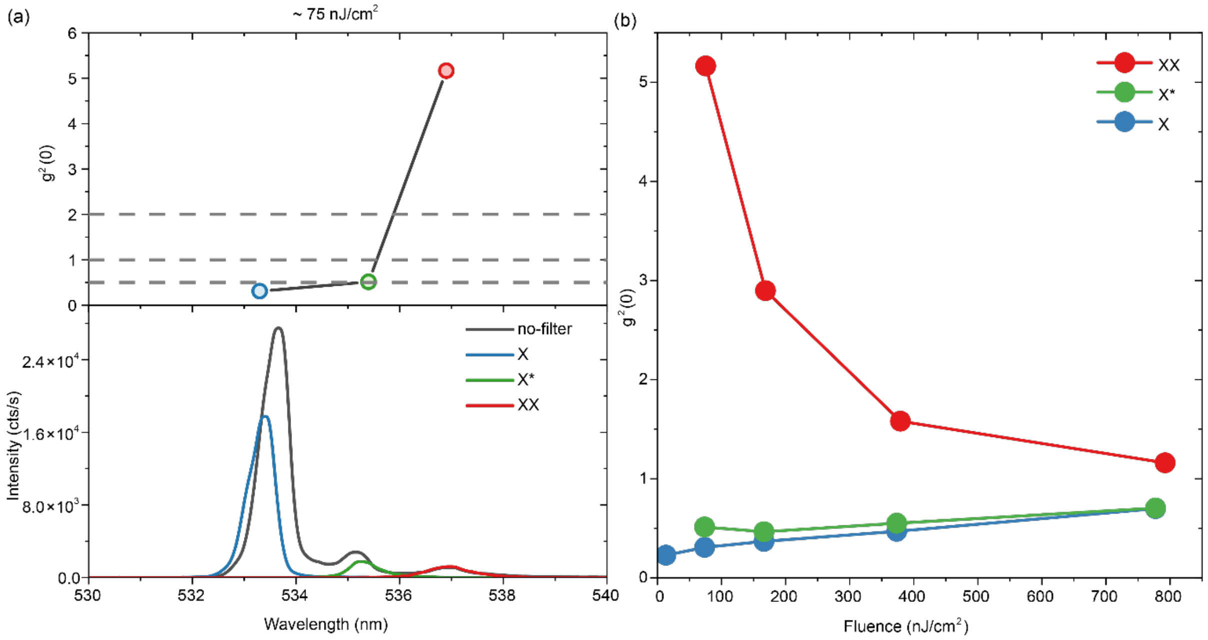

Figure S7. Spectrally resolved $g^{(2)}(0)$ values at the spectral region of exciton (X), trion (X*) and biexciton (XX). (a) $g^{(2)}(0)$ values at the spectral region of X, X* and XX at an excitation fluence of around 75 nJ/cm^2. (b) Excitation-fluence-dependent $g^{(2)}(0)$ values at the spectral region of X, X* and XX.

In the real experiment, at an excitation density of $\langle n \rangle = 0.05$ per excitation pulse where we obtained the bunching peak with a $g^{(2)}(0)$~7 (see Figure 4), we detect 208 energy-degenerate photon pairs on the 50/50 beam splitter over a 100 s integration time, see Figure R1. In comparison, a Poissonian source with the same average photon counts would theoretically produce only about 28 photon pairs.

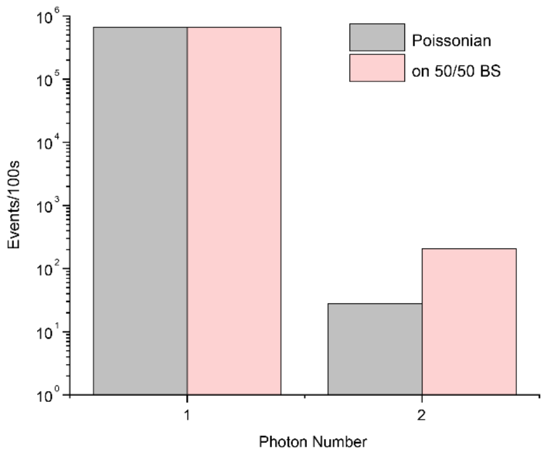

Figure S8. Number of detected events of a single photon (photon number = 1) and photon pairs (photon number = 2) when employing our QD source (red) or considering a Poissonian distribution with the same average photon counts (grey). For our QD source (red), the employed excitation density is $\langle n \rangle = 0.05$ per excitation pulse and the signal was acquired for 100 s.

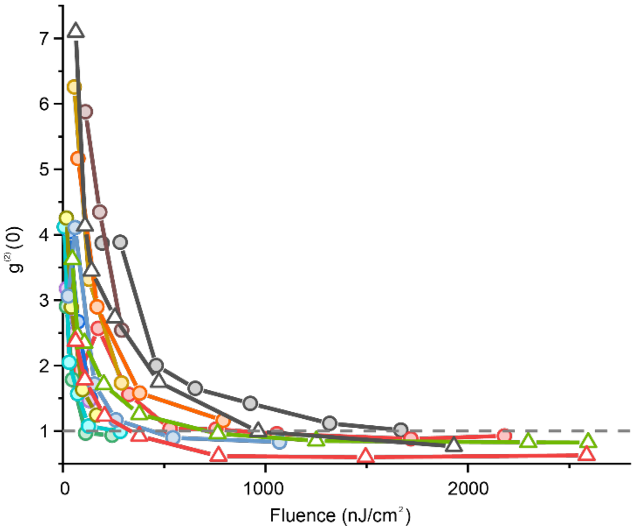

Figure S9. Excitation-dependent $g^{(2)}(0)$ at the spectral region of biexciton in different QDs.

4. Numerical calculation of the photon statistics

The probability of generating n excitons by absorbing on average $\langle n \rangle$ excitons per excitation pulse is

$$p(n) = \frac{\langle n \rangle^{n} e^{-\langle n \rangle}}{n!} \quad (S5)$$

where $\langle n \rangle$ can be calculated based on the excitation density in the focal plane, the intrinsic absorption cross section[5] of $CsPbBr_3$, and the mean QD size in the ensemble (see TEM images in Figure S1).

Then, in the auto-correlation function, $g^{(2)}(0) = \frac{center}{side}$ with

$$center = \alpha\beta 2 \sum_{m=2}^{\infty} p(n \geq m) \sum_{m'=1}^{m-1} \eta_m \eta_{m'} \quad (S6)$$

and

$$side = \alpha\beta (\sum_{m=1}^{\infty} p(n \geq m) \eta_m)^2 \quad (S7)$$

which are adapted from Ref.[6] with α and β being the detection probabilities of an emitted photon, including all the losses from the QDs emission to the detectors, η_m representing the probability of the one photon emitted from the transition of $|m\rangle$ to $|m-1\rangle$. When calculating the $g^{(2)}(0)$ values of the photon pairs of biexciton and OP_3 phonon, we assume $\eta_1 = 0.01$ based on the coupling strength of this phonon mode (see Figure S6).

References

(1) Akkerman, Q. A.; Nguyen, T. P.; Boehme, S. C.; Montanarella, F.; Dirin, D. N.; Wechsler, P.; Beiglböck, F.; Rainò, G.; Erni, R.; Katan, C. Controlling the nucleation and growth kinetics of lead halide perovskite quantum dots. *Science* **2022**, *377* (6613), 1406-1412.

(2) Yang, J.; Hugonin, J.-P.; Lalanne, P. Near-to-far field transformations for radiative and guided waves. *ACS Photonics* **2016**, *3* (3), 395-402.

(3) Becker, M. A.; Vaxenburg, R.; Nedelcu, G.; Sercel, P. C.; Shabaev, A.; Mehl, M. J.; Michopoulos, J. G.; Lambrakos, S. G.; Bernstein, N.; Lyons, J. L. Bright triplet excitons in caesium lead halide perovskites. *Nature* **2018**, *553* (7687), 189-193.

(4) Zhu, C.; Feld, L. G.; Svyrydenko, M.; Cherniukh, I.; Dirin, D. N.; Bodnarchuk, M. I.; Wood, V.; Yazdani, N.; Boehme, S. C.; Kovalenko, M. V. Quantifying the size‐dependent exciton‐phonon coupling strength in single lead‐halide perovskite quantum dots. *Adv. Opt. Mater.* **2024**, *12*, 2301534.

(5) Maes, J.; Balcaen, L.; Drijvers, E.; Zhao, Q.; De Roo, J.; Vantomme, A.; Vanhaecke, F.; Geiregat, P.; Hens, Z. Light absorption coefficient of $CsPbBr_3$ perovskite nanocrystals. *J. Phys. Chem. Lett.* **2018**, *9* (11), 3093-3097.

(6) Nair, G.; Zhao, J.; Bawendi, M. G. Biexciton quantum yield of single semiconductor nanocrystals from photon statistics. *Nano Lett.* **2011**, *11* (3), 1136-1140.
